# Supplementary material for: Genome-wide CRISPR Screens in T Helper Cells Reveal Pervasive Crosstalk between Activation and Differentiation
Source: Cell. 2019 Feb 7;176(4):882–896.e18. doi: 10.1016/j.cell.2018.11.044 (PMC6370901; doi:10.1016/j.cell.2018.11.044)
Supplement: Data S2. Processed Data from All the Steps of the Analysis, Related to Figure 1 [file mmc2.zip › supplemental data/motif analysis/Bhlhe40_homer/homerResults/motif12.similar.html]

motif12

## Information for motif12

A
C
G
T
A
G
T
C
C
A
G
T
A
C
T
G
T
G
A
C
C
A
T
G
C
G
T
A
C
T
G
A
T
G
A
C
A
G
T
C
  
Reverse Opposite:  

A
C
T
G
A
C
T
G
A
G
C
T
A
C
G
T
G
T
A
C
A
C
T
G
A
G
T
C
G
T
C
A
A
C
T
G
G
T
C
A
  

|  |  |
| --- | --- |
| p-value: | 1e-10 |
| log p-value: | -2.439e+01 |
| Information Content per bp: | 1.841 |
| Number of Target Sequences with motif | 22.0 |
| Percentage of Target Sequences with motif | 1.83% |
| Number of Background Sequences with motif | 124.5 |
| Percentage of Background Sequences with motif | 0.29% |
| Average Position of motif in Targets | 105.0 +/- 50.5bp |
| Average Position of motif in Background | 106.8 +/- 60.8bp |
| Strand Bias (log2 ratio + to - strand density) | 0.3 |
| Multiplicity (# of sites on avg that occur together) | 1.00 |
| Motif File: | file (matrix) reverse opposite |

### Similar de novo motifs found

|  |  |  |  |  |  |  |  |
| --- | --- | --- | --- | --- | --- | --- | --- |
| Rank | Match Score | Redundant Motif | P-value | log P-value | % of Targets | % of Background | Motif file |
| 1 | 0.653 | A C G T C G A T A G T C A C G T A T C G T G C A T C A G C G T A | 1e-4 | -11.393305 | 2.33% | 0.92% | motif file (matrix) |
